# Primary care: a fading jewel in the NHS crown

**DOI:** 10.1080/17571472.2015.1082343

**Published:** 2015-09-28

**Authors:** Azeem Majeed

**Affiliations:** ^a^ Department of Primary Care & Public Health, Imperial College London, London, UK; ^b^ Clapham Manor Health Centre, Lambeth CCG, London, UK

**Keywords:** Primary care, NHS, England

## Abstract

When Jeremy Hunt, the Secretary for State for Health in England, presented his ‘New Deal for General Practice’ in June 2015, he described general practice as the jewel in the crown of the NHS. Many general practitioners (GPs) though will not be reassured by his statement. Despite Jeremy Hunt’s words of support, the future for GPs, their teams and their patients looks very uncertain. It is hard to see how planned levels of funding for the NHS in England can sustain a readily accessible, high-quality primary care service. It seems likely that primary care in England will increasingly be delivered by non-medical professionals, such as pharmacists, nurses, physician assistants and health care assistants. The acceptability to patients – and the impact on quality of care, patient outcomes and the other parts of the NHS – of this model are all unknown. An alternative scenario is that we gradually move to a ‘two-tier’ primary care system with those patients who can afford to do so paying to see a medically qualified GP.

## Why this matters to me

I have worked as an academic general practitioner (GP) in London for 20 years. As an academic GP, teaching, training and research in primary care are important parts of my work. The future of general practice in England looks very uncertain and there are increasing problems in training, recruiting and retaining GPs. I am concerned that these problems will start to have an adverse effect on the NHS, and on access to high-quality primary care services.

## Key messages

• The future for GPs in England, their teams and their patients looks very uncertain.
• It is hard to see how planned levels of funding for the NHS in England can sustain a readily accessible, high-quality primary care service.
• It seems likely that primary care in England will increasingly be delivered by non-medical professionals.
• The acceptability to patients – and the impact on quality of care, patient outcomes and the other parts of the NHS – of this model are all unknown.

When Jeremy Hunt, the Secretary for State for Health in England, presented his ‘New Deal for General Practice’ in June 2015, he described general practice as the jewel in the crown of the NHS. Many GPs though will not be reassured by his statement. Despite Jeremy Hunt’s words of support, general practice in the NHS has been very shabbily treated by the Department of Health and NHS England in recent years and it is now very much a fading jewel.

**Figure 1.**
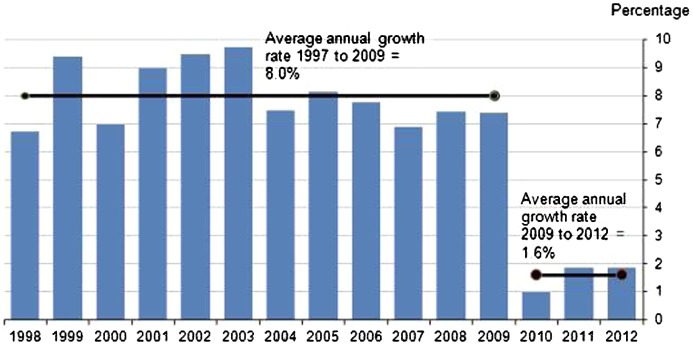
Annual growth in NHS spending in the UK, 1997–2012.

Source: Office for National Statistics.

**Figure 2.**
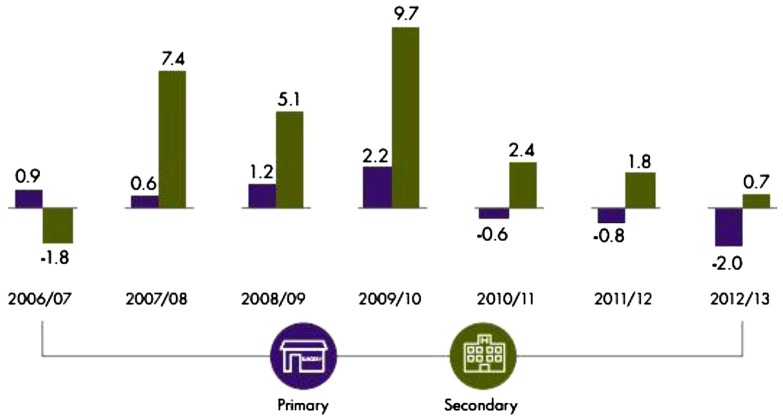
Annual real terms percentage change in spending on primary and secondary care in England since 2006/2007

Source: Primary care trust annual accounts data, 2006/2007 to 2012/2013 (via Nuffield Trust).

**Figure 3.**
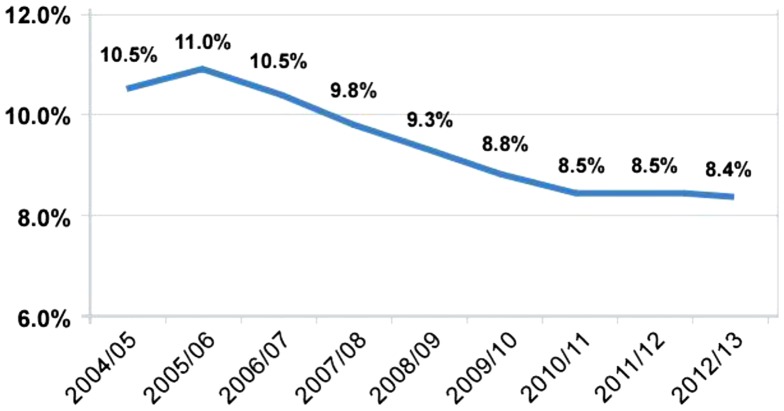
GP funding as a share of NHS expenditure in England, 2004/2005 – 2012/2013.

Source: Royal College of General Practitioners.

**Figure 4.**
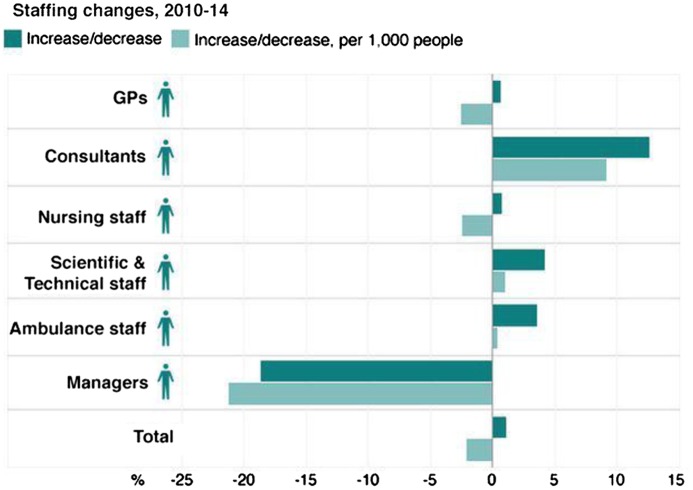
NHS staffing changes in England, 2010–2014.

Source: King’s Fund.

The most pressing problems facing general practices are their funding and workload, along with the recruitment and retention of GPs. The UK Office for National Statistics (ONS) reports that NHS funding grew by an average of 8.0% annually during the period from 1997 to 2009. During the period 2009–2012, the annual growth in NHS spending then fell to 1.6% (Figure 1). When the Nuffield Trust examined changes in NHS spending between primary care and secondary care, they reported that primary care had experienced a much lower growth in spending than the hospital sector in recent years (Figure 2). The effect of these changes has been to reduce the proportion of the NHS budget spent on general practice from 11.0% in 2005/2006 to just 8.4% by 2012/13 (Figure 3). Unsurprisingly, the growth in the number of GPs in England has therefore been much lower than that of hospital consultants in recent years (Figure 4).

With an increasing number of NHS hospital trusts reporting financial problems, it seems unlikely that NHS England will make any significant attempt in the foreseeable future to move funding from the hospital sector to primary care as to do so would further destabilise many hospitals. Furthermore, as the Government remains committed to reducing its budget deficit, NHS spending is unlikely to increase very much in real terms during the course of this parliament. This means that general practices will not see very much improvement in their financial situation in the foreseeable future.

To compound their funding problems, many GPs also report steadily rising levels of workload and higher patient expectations. Because the NHS does not collect or publish data on GPs’ workload (unlike the hospital sector where we see statistics on workload published regularly), it is impossible to say with any accuracy by how much the volume and intensity of their work has increased in recent years. We are though beginning to see a larger proportion of patients reporting problems in getting appointments with their GPs. General practitioners’ morale is also being affected. In a BMA Survey published in July 2015, over 70% of GPs reported that their current workload was unmanageable or unsustainable.

Funding and workload problems are in turn affecting recruitment and retention of GPs. A high proportion of GPs are aged over 55 years and due to retire in the next decade. At the other end of the retention and recruitment ladder, many specialist general practice training schemes are reporting difficulties in filling their training places. Newly qualified GPs are often opting to work as locums or overseas rather than take up established posts. Consequently, it is increasingly difficult to recruit GPs in many parts of England, imposing further strains on the existing primary care workforce.

What does the future hold for general practice in England? We have seen a number of new initiatives to support primary care announced by the Department of Health and NHS England during the summer of 2015. But these are little more than tokenistic ‘sticking plaster solutions’ that will not address the underlying problems that primary care is currently experiencing. If GPs cannot be recruited and trained in sufficient numbers, it seems likely that primary care in England will increasingly be delivered by non-medical professionals such as pharmacists, nurses, physician assistants and health care assistants.

Some commentators such as Richard Smith, the former editor of the British Medical Journal, have argued that this model of delivering primary care, relying increasingly on non-medical professionals, would be a positive development for the NHS. But the acceptability to patients – and the impact on quality of care, patient outcomes and the other parts of the NHS – of this model are all unknown. An alternative scenario is that we gradually move to a ‘two-tier’ primary care system with those patients who can afford to do so paying top-up fees to see a medically qualified GP and the less well-off seeing a range of non-medical professionals.

In conclusion, the future for GPs, their teams and their patients looks very uncertain. It is hard to see how current levels of funding can sustain a readily accessible, high-quality service during the period Monday to Friday. Trying to force general practices to extend themselves to provide a ‘7 day service’ may well result in increasing problems with the delivery of services from Monday to Friday. Either politicians and the public will have to be more realistic about what the NHS can offer with its current level of funding or we will need to consider the introduction of charges for using primary care services in an attempt to control demand and to provide sufficient funding for the services that people expect from their GPs.

